# A Kernel-Based Multivariate Feature Selection Method for Microarray Data Classification

**DOI:** 10.1371/journal.pone.0102541

**Published:** 2014-07-21

**Authors:** Shiquan Sun, Qinke Peng, Adnan Shakoor

**Affiliations:** Systems Engineering Institute, School of Electronic and Information Engineering, Xi'an Jiaotong University, Xi'an, China; University of Westminster, United Kingdom

## Abstract

High dimensionality and small sample sizes, and their inherent risk of overfitting, pose great challenges for constructing efficient classifiers in microarray data classification. Therefore a feature selection technique should be conducted prior to data classification to enhance prediction performance. In general, filter methods can be considered as principal or auxiliary selection mechanism because of their simplicity, scalability, and low computational complexity. However, a series of trivial examples show that filter methods result in less accurate performance because they ignore the dependencies of features. Although few publications have devoted their attention to reveal the relationship of features by multivariate-based methods, these methods describe relationships among features only by linear methods. While simple linear combination relationship restrict the improvement in performance. In this paper, we used kernel method to discover inherent nonlinear correlations among features as well as between feature and target. Moreover, the number of orthogonal components was determined by kernel Fishers linear discriminant analysis (FLDA) in a self-adaptive manner rather than by manual parameter settings. In order to reveal the effectiveness of our method we performed several experiments and compared the results between our method and other competitive multivariate-based features selectors. In our comparison, we used two classifiers (support vector machine, 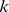-nearest neighbor) on two group datasets, namely two-class and multi-class datasets. Experimental results demonstrate that the performance of our method is better than others, especially on three hard-classify datasets, namely Wang's Breast Cancer, Gordon's Lung Adenocarcinoma and Pomeroy's Medulloblastoma.

## Introduction

Microarray gene expression based cancer classification is one of the most important tasks in bioinformatics. A typical classification task is to separate healthy patients from cancer patients, based on their gene expression “profile”. However, because cancers are usually marked by changing in the expression levels of certain genes [1], therefore it is obvious that not all measured features are discriminative features for target. Hence, feature selection problem is ubiquitous in cancer classification.

Feature selection techniques for microarray data can be broadly grouped into three categories that are wrapper (classifier-dependent) methods [2], [3], embedded (classifier-dependent) methods [4], [5] and filter (classifier-independent) methods [6], [7]. The primary distinguishing factors among them are computational complexity and the chance of overfitting [8]. Generally, in terms of computational cost, filters are faster than embedded methods, which are in turn faster than wrappers. In terms of overfitting, wrappers have higher learning capacity so are more likely to overfit than embedded methods, which in turn are more likely to overfit than filter methods [9]. Filter methods can be divided into two classes, univariate-based filters and multivariate-based filters [8]. Univariate filter methods have attracted much attention because of their low complexity and fast performance for high dimensionality of microarray data analyses. However, some valuable genes discarded by univariate methods may have great contribution for classification [10]. Therefore, the major reason of their less accurate performance is that they disregard the effects of feature-feature(we use without distinction the term “feature” and “gene” in the paper) interactions. The applications of multivariate filter methods are simple bivariate-based methods which are almost based on entropy(or conditional entropy) and mutual information [9], [11], such as mRMR [7], [12], CFS [13] and several variants of the Markov blanket filter method [14]. However, they also abandon presumably redundant variables that can result in a performance loss [15].

Partial least squares(denoted as PLS), which shares the characteristics of other regression and feature transformation techniques(such as canonical correlation analysis and principal component analysis), has proven to be useful in situations when the number of observed variables(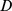) are significantly greater than the number of observations(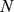) (e.g.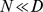). In other words, PLS is a popular approach to solve problems when there is high multicollinearity among features [16]. SlimPLS [17],PLSRFE [18], [19] and TotalPLS [20] are multivariate-based feature selection methods that were proposed by Gutkin et al. and You et al., respectively. Unfortunately, classical PLS technique is essentially a linear regression method that only can capture the linear relationships between genes in original space. In real biological applications, linear relationship often fails to fully capture all the information among genes. Kernel method, which approaches the problem by projecting the data into a high dimensional feature space, is commonly used for revealing the intrinsic relationships that are hidden in the raw data.

Motivated by mentioned above, in this paper, we develop a feature selection method based on the partial least squares(abbreviated PLS) [21] and theory of *Reproducing Kernel Hilbert Space* [22], we called it *kernelPLS*(publicly available at https://github.com/sqsun/kernelPLS). Determining the number of components is a thorny problem in PLS(also in kernelPLS) method. In order to obtain a reasonable number of components, we make use of the relationship between PLS and linear discriminant analysis to determine the number of components in kernel space based on kernel linear discriminant analysis. We find that the two classifiers combined with our feature selection method obtained promising classification accuracy on eleven microarray gene expression datasets.

The rest of this paper is organized as follows. In section 2 we proposed a filter method based on PLS and kernel method. Then we proceed in section 3 to determine the optimal parameters for our method. In section 4 we compared our approach with several competitive filters. The conclusion can be found in section 5.

## Methods

In the following, let 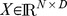 represents a data matrix of 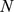 inputs (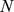 samples) and 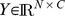 stands for corresponding response matrix of 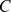-dimensional(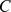 classes). Further we assume columns of 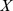 and 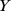 are zero-mean.

### Kernel partial least squares

PLS is one of the widespread use of a class of multivariate statistical analysis technique introduced by [21], and a popular regression technique in Chemometrics [23]. It differs from other methods in constructing the fundamental relations between two matrices (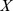 and 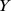) by means of latent variables called *components*, leading to a parsimonious model which shared characteristics with other regression and feature transformation techniques [16]. The goal of PLS is to calculate vectors of its 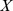-weight (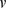), 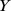-weight (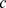), 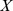-score (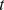) and 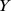-score (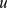) by an iterative method for the optimization problem: 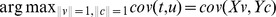. Where 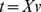 and 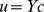, are called components of 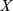 and 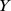, respectively.

**Figure 1:**
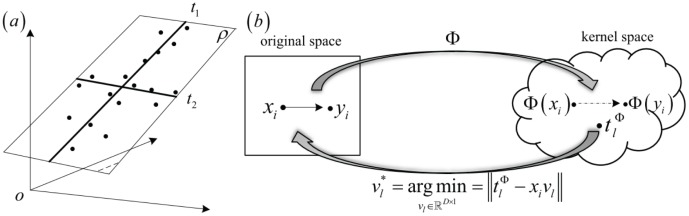
The geometric representation of PLS and kernel PLS. (a) In the original space, the components 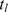, 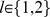 are on plane 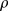. (b) We projected the data into the kernel space by mapping 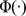 and the components 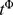 are captured in kernel space. The weight of each feature is estimated by 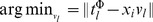, 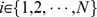.

When the first two components 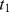 and 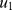 are obtained, the second pair 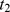 and 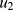 is extracted from their residuals 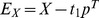 and 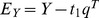, respectively. Here 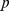 and 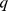 are called the *loadings* of 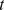 with respect to 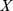 and 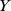, respectively. This process can be repeated until the required halt condition is satisfied. The detail description of the algorithm can be found in [17]. The geometric representation of PLS can be found in Figure 1(a).

The kernel version of PLS uses a nonlinear transformation 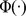 to map the gene expression data into a higher-dimensional(even infinite dimensional) kernel space 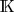; i.e. mapping 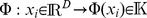. However, we do not need to know the specific mathematical expression of nonlinear mapping, we only need to state the entire algorithm in terms of dot products between pairs of inputs and substitute kernel function 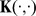 for it. This is so-called the “*kernel trick*”.

In order to state dot product operation in the algorithm, we can restrict 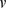 to belong to the linear spans of the points. They can therefore be expressed as:
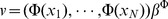


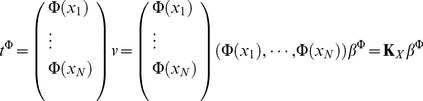



Let 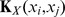 be an element of the *Gram matrix* 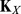 in feature space and 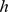 is the desired number of components. Deflating 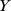 will, however, be needed for kernel partial least squares.

The first component for kernel PLS can be determined as eigenvector of the following square kernel matrix for 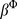: 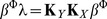, where 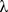 is an eigenvalue. The size of the kernel matrix 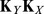 is 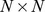. Hence, no matter how many variables there are in the original matrices 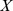 and 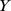, the size of these kernel matrices will not be get affected by it. Therefore, the combination of PLS with kernel produces a powerful algorithm that will solve this problem rapidly and effectively. The geometric representation of kernel PLS can be found in Figure 1(b). The kernel PLS algorithm procedure and the number of determined components can be found in Table 1 (https://github.com/sqsun/kernelPLS).

**Table 1:** Algorithm 1: kernelPLS.

**Input**: 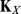 – kernel matrix
_ _ 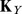 – kernel matrix
**Output**: 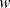 – the weight of each feature
1: Initializing 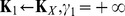 ;
_ _ 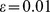 , 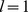 ;
2: **while** 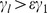 **do**
3: Initializing the projection direction 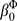 , 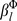 ;
4: **while ** 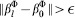 **do**
5: 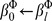 ;
6: 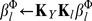 ;
7: 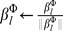 ;
8: **end while**
9: Calculating the component 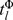 , 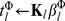 ;
10: Deflating target matrix 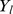 , 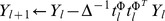 , where 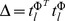 ;
11: Deflating kernel matrix 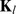 , 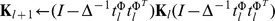 ;
12: Calculating the contribution of the 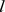 th component 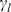 , 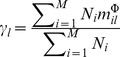 ;
13: 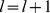 ;
14: **end while**
15: 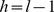
16: Calculating the weight of each feature 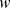 via Equation(1)
17: **return ** 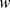

### The importance of each feature

In original space, let 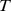 is a set of components, 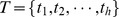. The accumulation of variation explanation of 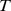 to 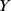 is given by [24], [25]

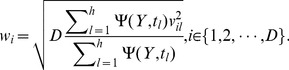
(1)where 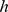 is the number of components and 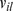 is the weight of the 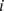
th feature for the 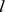
th component. 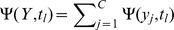 is the correlation between 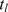 and 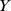, where 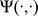 is correlation function. The larger value of 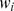, the more explanatory power of the 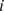
th feature to 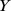.

It is worth noting that the above equation can also be used in kernel space. The reason is holding of equation 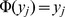, because here 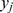 is class label. So the expression 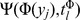 can be expressed as 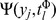, here 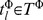 and 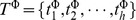.

### Model selection

Two issues are still unresolved before applying kernel PLS for feature selection. The number of components and the number of features are unknow.

### The number of components

In order to determine the number of components 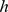, there are two widely used methods in the previous works, one is setting a fixed number, such as 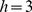, and another is by cross validation (CV). Different datasets contain various data structures, therefore, a fixed number is not suitable for all datasets. Although the CV combined with various classifiers lead to good performance, it suffers from huge computational burden.

To fully circumvent these difficulties, [26] has given an implication of close relationship between PLS and Fisher's linear discriminant analysis (FLDA) in original space. FLDA can be considered as an optimization problem 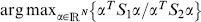, e.g. finding an appropriate projection vector 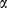. Where 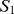 presents the inter-class scatter matrix, 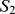 denotes the intra-class scatter matrix.

In kernel space, the FLDA turns out to be an optimization problem 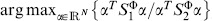, where 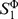 and 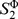 are the inter-class scatter matrix and the intra-class scatter matrix in kernel space, respectively. We consider
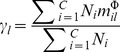



It denotes the contribution of the 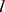
th component for classification. Where 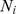 indicates the number of samples in the 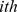 class, here 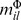 represents the mean vector of the 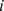 th class with respect to 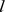 th component in projection space and the 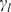 represents segmentation threshold of classification, the larger 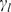 corresponds to the more significant in classification.

### The number of features

**Figure 2:**
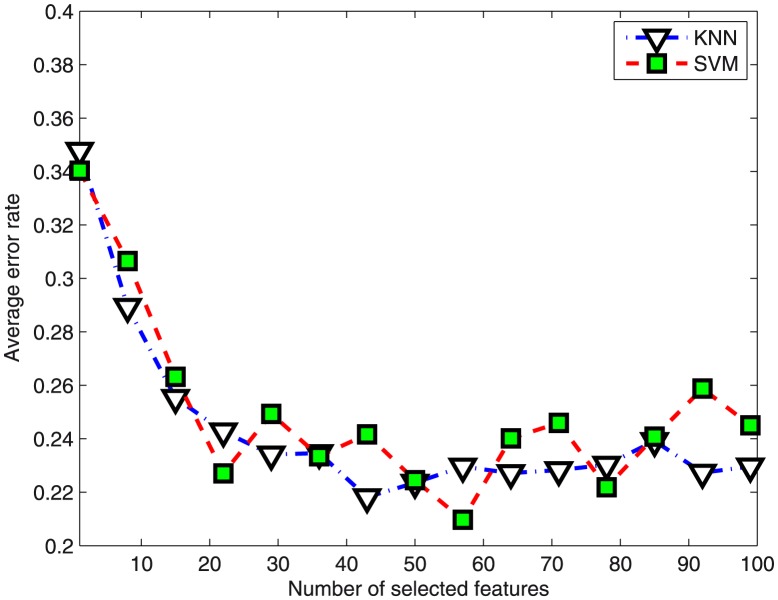
The effect of different numbers of selected features. Two classifier, SVM and KNN, are used for measuring the performance of average error of all test datasets based on kernelPLS selector. Where the optimal parameters of RBF kernel SVM are determined by partial swarm optimization and the parameter 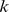 for the nearest neighbors is 5.

Figure 2 shows how classification performance varies with the change in number of features which were selected. The average classification error rate was calculated by two classifiers on all test datasets. An improvement in performance could be evident if the number of related features increase from 1 to 25, but after increasing number of features beyond 25, no significant improvement was obvious. In order to find optimum results for all the datasets, we extend the range from 20 to 50 features configurations in our study.

## Results

### Test datasets

To assess the performance of our method, we have conducted several experiments on a number of publicly available datasets. Summary of all data sets we used in our experiments can be found in table 2 and following is the brief description of each data set.

**Table 2:** The cancer classification datasets^1^ used in the present paper.

Class	Dataset	Sample	Feature	Class	Source
Two-class	AMLALL	72	7129	2	[27]
	Breast	209	22283	2	[42]
	Lung	86	7129	2	[29]
	Prostate	102	12600	2	[30]
	DLBCL	77	7129	2	[31]
	Medulloblastoma	60	7129	2	[32]
Multi-class	Stjude	215	12558	7	[13]
	Lymphoma	62	4026	3	[33]
	SRBCT	83	2308	4	[34]
	MLL	72	8685	3	[35]
	Lung	203	3312	5	[37]

1 Available at https://github.com/sqsun/kernelPLS-datasets.

- **AMLALL(A)([27]**).**** There are two parts containing the initial (train), 38 bone marrow samples from two classes: 27 cases of acute lymhoblastic leukemia(ALL) and 11 cases of acute myeloid leukemia(AML); independent (test), 34 samples from two classes: 20 cases of ALL and 14 cases of AML. Each case is described by expression levels of 7129 probes from 6817 human genes. Source: http://www-genome.wi.mit.edu/cgi-bin/cancer/datasets.cgi;
- **Breast(B)([28]**).**** The dataset used the raw intensity Affym-etrix CEL files and normalized the data by RMA procedures. A final expression matrix comprising 22283 features and 209 samples, 71 of which are from patients, the rest 138 samples are normal samples. Source: http://math.bu.edu/people/sray/software/prediction;
- **Lung(L)([29]** **).** This dataset contains 86 samples: 24 are tumor samples and 62 are normal controls, 7129 genes with highest intensity across the samples are considered. Source: http://math.bu.edu/people/sray/software/prediction/;
- **Prostate(P) ([30]** **).** This dataset contains 52 prostate tumor samples and 50 normal samples with 12600 genes. An independent set of testing samples is generated from the training data, 25 tumor and 9 normal samples are extracted according to Singh's publication. Source (training): http://www.broadinstitute.org/cgi-bin/cancer/datasets.cgi;
- **DLBCL(D)([31]** **).** The goal of this dataset is to distinguish diffuse large B-cell lymphoma (DLBCL) from follicular lymphoma (FL) morphology. This dataset contains 58 DLBCL samples and 19 FL samples. The expression profile contains 7129 genes. Source: http://www-genome.wi.mit.edu/mpr/prostate;
- **Medulloblastoma(M)([32]** **).** Patients outcome prediction for central nervous system embryonal tumor. Survivors are patients who are alive after treatment whiles the failures are those who succumbed to their disease. The dataset contains 60 patient samples, 21 are survivors and 39 are failures. There are 7129 genes in the dataset. Source: http://www-genome.wi.mit.edu/mpr/CNS;
- **Stjude(S)([13]** **).** The dataset has been divided into six diagnostic groups, BCR-ABL (9 samples), E2A-PBX1 (18 samples), Hyperdiploid
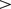
50 (42 samples), MLL (14 samples), T-ALL (28 samples) and TEL-AML1 (52 samples)), and one that contains diagnostic samples (52 samples) that did not fit into any one of the above groups. There are 12558 genes. Source: http://www.stjuderesearch.org/data/ALL1;
- **Lymphoma(Ly)([33]** **).** The dataset consists of measurements of 4026 genes from 62 patients. The patients are classified into three classes: lymphoma and leukemia (DLCL, 42 samples), follicular lymphoma (FL, 9 samples) and chronic lymphocytic leukemia (CLL, 11 samples). We estimated the missing values of “NA” symbol in original ratio data by KNN-imputed method (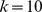). Source: http://llmpp.nih.gov/lymphoma;
- **SRBCT(SR)([34]** **).** The dataset contains 83 samples and 2,308 gene expression values. It can be divided into four classes, the Ewing family of tumors (EWS), Burkitt lymphoma(BL), neuroblastoma (NB) and rhabdomyosarcoma (RMS). Among the 83 samples, 29, 11, 18, and 25 samples belong to classes EWS, BL, NB and RMS, respectively. Source: http://www.biomedcentral.com/content/supplementary/1471-2105-7-228-S4.tgz.
- **MLL(ML)([35]** **).** The dataset contains 72 samples in three classes, acute lymphoblastic leukemia (ALL), acute myeloid leukemia (AML), and mixed-lineage leukemia gene (MLL), which have 24, 28, 20 samples, respectively. In our experiment, we preprocessed this dataset according to reference [36] and obtained a dataset with 72 samples and 8685 genes. Source: http://www.biomedcentral.com/content/supplementary/1471-2105-7-228-S4.tgz.
- **Lung(Lu)([37]** **).** The total of this dataset contains 203 samples with 12600 genes in five classes, adenocarcinomas (139), squamous cell lung carcinomas (21), pulmonary carcinoids (20), small-cell lung carcinomas(6) and normal lung (17). We preprocessed the dataset according to reference [36] and obtained a dataset with 203 samples and 3312 genes. Source: http://www.biomedcentral.com/content/supplementary/1471-2105-7-228-S4.tgz.

### Comparison of selected genes

In our first experiment, we used two datasets, namely the Leukemia data (two-class) of [27] and the Lymphoma data(three-class) of [33], to compare our method with previous works with respect to the selected genes.

For the Leukemia data, we collected several most important genes (in table 3) that were published in several papers. It can readily be seen that three probes, X95735_at, M27891_at and M23197_at were reported by five published papers, and their ranking by our method are 4th, 17st and 8st, respectively. We notice that there are many overlapping of genes among the list of papers.

**Table 3:** Description of genes reported by existing published papers and ranked by our method.

Accession number	Gene description	References	Rank
X95735_at	Zyxin	[43] [38] [27] [44] [28]	4
M23197_at	CD33	[43] [38] [27] [44] [28]	8
U22376_cds2_s_at	C-myb	[38] [27] [44] [28]	74
M27891_at	Cystatin C	[43] [38] [27] [44] [28]	21
M16038_at	LYN	[38] [27] [44] [28]	11
M84526_at	DF(adipsin)	[43] [38] [27] [44]	9
M27783_s_at	ELA2 Elastatse 2	[38] [44] [28]	80
U50136_rna1_at	LTC4 synthase	[38] [27] [28]	3
Y12670_at	Leptin receptor	[38] [27] [28]	2
U46499_at	Glutathione	[43] [38] [44]	96
L09209_s_at	Amyloid beta	[43] [38] [44]	48
U46751_at	p62	[38] [27]	19
M55150_at	Fumarylacetoacetate	[38] [27]	7
M83652_s_at	Properdin	[38] [27]	22
M80254_at	CyP3	[27] [28]	17
X17042_at	Proteoglycan 1	[43] [27]	10
U82759_at	HoxA9	[43] [27]	8

For Leukemia data, the top-ranked 40 features obtained by our procedure are shown in table 4 in which genes are in columns from 1 to 40. There is a worthwhile result achieved by our method, that is, it obtained the genes with the highest weight. Many of these genes are known as differentially expressed genes by many foregoing studies. 24 out of 40 genes are listed in this table that were also selected by [27], which shows the effectiveness of our method.

**Table 4:** Top-ranked 40 features selected using kernelPLS for the Leukemia dataset.

1. **M23197_at** 1	11.**M16038_at**	21.**M27891_at**	31.**M28130_rna1_s_at**
2.**Y12670_at**	12.**M96326_rna1_at**	22.**M83652_s_at**	32.M37435_at
3.**U50136_rna1_at**	13.X70297_at	23.M19507_at	33.M98399_s_at
4.**X95735_at**	14.**M62762_at**	24.**M63138_at**	34.U12471_cds1_at
5.D49950_at	15.**X85116_rna1_s_at**	25.X58431_rna2_s_at	35.U37055_rna1_s_at
6.**X04085_rna1_at**	16.**L08246_at**	26.**Y00787_s_at**	36.U67963_at
7.**M55150_at**	17.**M80254_at**	27.M68891_at	37.Y07604_at
8.**U82759_at**	18.M22960_at	28.X52056_at	38.**M69043_at**
9.**M84526_at**	19.**U46751_at**	29.M11147_at	39.U63289_at
10.**X17042_at**	20.M81933_at	30.**M57710_at**	40.**M81695_s_at**

1 The boldfaced probes were selected by [27].

For the Lymphoma data of [33], the missing values are imputed by KNN-imputed method(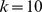). The top 40 genes ranked by our procedure are listed in table 5. From the table, We can see that important genes can be captured easily by our method. There are many genes that are also chosen by [38].

**Table 5:** Top-ranked 40 features selected using kernelPLS for the Lymphoma dataset.

1.**GENE1622X** 1	11.GENE1608X	21.GENE1636X	31.GENE1646X
2.**GENE2403X**	12.**GENE622X**	22.GENE710X	32.GENE721X
3.**GENE653X**	13.**GENE833X**	23.GENE2401X	33.GENE709X
4.**GENE1644X**	14.**GENE712X**	24.GENE1641X	34.GENE699X
5.GENE1607X	15.GENE735X	25.GENE654X	35.GENE2110X
6.**GENE1647X**	16.**GENE1553X**	26.GENE1661X	36.GENE639X
7.**GENE1610X**	17.GENE708X	27.**GENE1702X**	37.GENE717X
8.**GENE2402X**	18.**GENE530X**	28.GENE642X	38.GENE2109X
9.GENE1648X	19.GENE675X	29.**GENE1744X**	39.GENE2399X
10.**GENE1643X**	20.**GENE2400X**	30.GENE689X	40.GENE2397X

1 The boldfaced genes were selected by [38].

**Figure 3:**
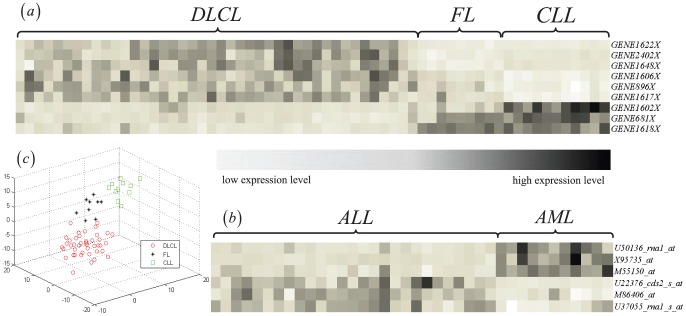
The genes expression levels of two datasets, namely the Leukemia and the Lymphoma data. Expression levels for each gene are normalized across the samples such that the mean is 0 and the SD is 1. Expression levels greater than the mean are shaded in black, and those below the mean are shaded in white. (a) The expression profiles of the Lymphoma dataset. Each row corresponds to a gene, with the columns corresponding to expression levels in different samples. (b) The expression profiles of the Leukemia dataset. Each row expresses a probe while each column describes expression level in different samples. (c) Display the results on the Lymphoma dataset using sammon mapping. This projection expresses the gene expression levels of genes that perfectly separate the three types of Lymphoma subtypes, i.e. DLBCL, FL, and CLL.

Figure 3 illustrates the differentially expressed genes for two datasets, namely the Leukemia data and the Lymphoma data. No single gene is uniformly expressed across the class, all these genes as a group appear correlated with class which is illustrating the effectiveness of the Kernel PLS method. In Figure 3(a) the top panel is consist of three genes GENE1622X, GENE2402X and GENE1648X that are highly expressed in DLCL, middle panel is comprised of GENE1606X, GENE896X and GENE1617X that are highly expressed in DLCL but moderately expressed in FL. Bottom panel compose of three genes, namely GENE1602X,GENE681X and GENE1618X, are more highly expressed in CLL. In Figure 3(b) the top panel shows three probes highly express in AML and the bottom panel shows three probes more highly expression in ALL. The probe U377055_rna1_s_at was found by our method to distinguish AML from ALL. Figure 3(c) demonstrate the projected result of top 100 genes using sammon mapping which shows DLBCL, CLL, FL are very clear and the boundaries can be easily drawn.

### Comparison of several multivariate-based feature selectors

In our second experiment, we compared several feature selectors with our procedure based on two classifiers, SVM and KNN. In our experiments, we choose the RBF kernel for each dataset to perform classification. To determine the best values of 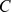
(-c) and 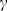
(-g), we conducted *particle swarm optimization* algorithm to pick the pair (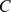,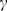) with best accuracy in the range of 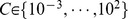 and 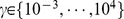. We set the parameter to 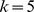 for 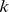-nearest neighbor. To obtain a statistically reliable predictive measurement, we performed 10-fold cross validation for two-class datasets and 5-fold cross validation for multi-class datasets. The results are evaluated by classification accuracy(Acc), area under receiver operating characteristic curve (AUC) for two-class problems and classification accuracy(Acc), Cohen's Kappa coefficient(Kappa) for multi-class problems. The reason of using 5-fold cross validation for multi-class datasets is that there is just a few number of samples in some groups (classes) of these datasets. Therefore to ensure the presence of samples of each class in training and also in test datasets we need to perform 5-fold cross validation for multi-class datasets.

In this paper, the comparison was conducted with four competitive algorithms, PLS, ReliefF, SVMrfe and mRMR. The parameter setting of them are as follows: for the PLS-based feature selection, we used the SIMPLS method and the number of components determined by self-adaptive manner which is the same as the kernelPLS (the proposed method). The parameter 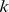 of ReliefF is equal to the number of sample according to the published paper [39]. For SVMrfe, in order to ensure acceptable running time, we use SVM with RBF kernel and its parameter settings are same as in LIBSVM.

**Figure 4:**
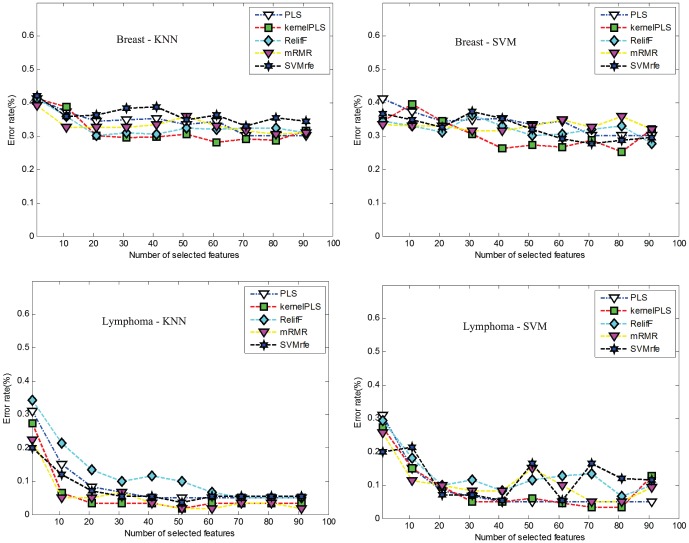
Classification error rate of different number of selected features using two classifiers, KNN and SVM. (a) and (b) indicate the results on the Breast dataset. (c) and (d) indicate the results on the Lymphoma dataset.

Without loss of generality, we used two datasets, Breast(two-class) and Lymphoma(three-class) to show the performance of our method. Figure 4 shows the comparison of error rate between our method and four other methods. One can see that when number of selected features are 30, error rate of our method is less than other methods for both classifiers and both datasets.

Table 6 and 7 summarized the comparison of results generated by our method and other methods with respect to Acc and AUC for two-class datasets. From the results, we can see that the performance of our method is better than others. Refers to table 6 we can see that for Breast(B) and Prostate(P) datasets, accuracy of our method is considerably higher as compare to other methods, which shows the effectiveness of our method.

**Table 6:** Comparison of kernelPLS with four other feature filters for the classification accuracy(%) and AUC(%) of KNN(k = 5) on two-class datasets.

Dataset	ReliefF		mRMR		SVMrfe		PLS		kernelPLS	
	Acc	AUC	Acc	AUC	Acc	AUC	Acc	AUC	Acc	AUC
A	96.1	98.3	97.5	98.3	**98.8**	**99.0**	90.7	97.3	94.6	**99.0**
B	68.5	66.5	67.9	67.7	68.5	67.2	69.9	70.2	**71.8**	**75.5**
L	74.2	77.4	74.2	74.2	74.3	75.5	**75.7**	76.5	73.2	**78.3**
D	93.8	97.5	**95.0**	**99.6**	93.4	98.4	91.1	96.3	**95.0**	97.1
M	70.0	73.5	71.7	77.3	65.0	68.8	73.3	**80.8**	**73.3**	76.2
P	95.0	98.1	**96.0**	96.6	90.1	92.3	95.3	98.3	**96.0**	**98.9**
Avg.	82.9	85.2	83.7	85.6	81.7	83.5	82.7	86.6	**84.0**	**87.5**

Similarly in table 7 for datasets Breast, Lung, DLBCL, Medulloblastoma, Prostate and Stjude, kernelPLS shown better accuracy rate for SVM classifier wrather than KNN. Both Acc and AUC values of our method have higher values among others and finally the average results likewise are best. Although for few datasets our results are similar to their results but in these cases time taken by our method is significantly smaller than other methods. For example in table 7 for AMLALL dataset, including our method, the AUC is 100% for many methods but time consumed by our method is only 0.0891 s while the time taken by other methods, ReliefF, mRMR, SVMrfe and PLS, are about 5 s, 52 s, 210 s and 12 s, respectively. So time consumption by our algorithm is many times less than others which depicts overall well performance of our method.

**Table 7:** Comparison of kernelPLS with four other methods. For 10-fold cross validation classification accuracy(%) and AUC (%) of SVM on two-class datasets.

Dataset	ReliefF		mRMR		SVMrfe		PLS		kernelPLS	
	Acc	AUC	Acc	AUC	Acc	AUC	Acc	AUC	Acc	AUC
A	**97.5**	**100**	96.3	**100**	**97.5**	**100**	94.6	**100**	96.1	**100**
B	68.0	69.2	69.9	67.5	69.9	69.7	72.2	71.5	**72.7**	**75.4**
L	**77.4**	81.5	72.1	76.5	73.3	75.8	76.8	77.6	**77.4**	**82.6**
D	94.8	99.2	94.8	99.2	93.4	99.4	93.4	98.3	**97.5**	**100**
M	71.7	72.9	70	73.1	66.7	69.7	70	77.2	**73.3**	**82.7**
P	96.0	97.5	96.0	96.7	89.1	94.2	95.1	**98.7**	**97.3**	**97.9**
Avg.	84.2	86.7	83.2	85.5	81.7	84.8	83.7	87.2	**85.7**	**89.8**

It is worth noting that our method outperforms others on three hard-classify datasets, Wang's Breast cancer, Gordon's Lung adenocarcinoma and Pomeroy's Medulloblastoma. We also make a comparison with the results of other feature selectors in published papers. Fox example, the reference [40] reported that the accuracies of 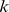-TSP+SVM on these datasets were 67.1%, 72.2% and 64.2%, respectively. The reference [41] combined multiple feature selection (or feature transform) approaches for Medulloblastoma dataset and the obtained highest Acc was 70%.

To estimate the performance of our method we did not limit our evaluation to only two-class datasets we also used 5 multi-class datasets in our experiments. Tables 8 and 9 demonstrate the comparison of kernelPLS with other methods for multi-class datasets on the bases of results obtained for two evaluation measures, namely Acc and Kappa. Results shown in table 8 and table 9 are for two classifiers KNN and SVM, respectively. In table 8 results obtained by kernelPLS are better than Relief, SVMrfe and PLS and highly competitive to mRMR method for several multi-class datasets. For example in case of Stjude dataset for Acc and Kappa values by kernelPLS are 96.4% and 0.956 respectively which are highest among all values achieved by other methods. Likewise table 9 authenticates the high performance by kernelPLS over other methods for SVM classifier. Here one can see that kernelPLS give outperforming results for all datasets by achieving accuracies and Kappa coefficients values superior than all other methods. As a conclusion the overall high average Acc and Kappa values in both tables show the effectiveness and significance of our method as compare to other popular methods.

**Table 8:** Comparison of kernelPLS with four other feature filters for the classification accuracy(%) and Cohen's kappa coefficient of KNN(k = 5) on multi-class datasets.

Dataset	ReliefF		mRMR		SVMrfe		PLS		kernelPLS	
	Acc	Kappa	Acc	Kappa	Acc	Kappa	Acc	Kappa	Acc	Kappa
St	83.9	0.811	88.7	0.852	81.9	0.797	86.9	0.842	**89.9**	**0.876**
Ly	98.5	0.964	**100**	**1**	98.3	0.969	**100**	**1**	**100**	**1**
Lu	72.2	0.271	73.3	0.403	73.3	0.268	76.8	0.404	**76.8**	**0.428**
ML	87.7	0.762	**94.6**	**0.903**	91.7	0.852	89.0	0.794	93.1	0.877
SR	91.6	0.884	**98.8**	**0.983**	91.5	0.880	91.5	0.877	96.4	0.947
Avg.	86.8	0.738	91.1	**0.828**	87.3	0.753	88.8	0.783	**91.2**	**0.826**

**Table 9:** Comparison of kernelPLS with four other methods. For 5-fold cross validation classification accuracy(%) and Cohen's kappa coefficient of SVM on multi-class datasets.

Dataset	ReliefF		mRMR		SVMrfe		PLS		kernelPLS	
	Acc	Kappa	Acc	Kappa	Acc	Kappa	Acc	Kappa	Acc	Kappa
St	86.2	0.849	88.9	0.866	86.4	0.851	86.8	0.834	**89.9**	**0.876**
Ly	**100**	**1**	**100**	**1**	96.7	0.933	**100**	**1**	**100**	**1**
Lu	76.9	0.451	76.9	0.399	74.6	0.382	74.5	0.360	**79.2**	**0.532**
ML	94.6	0.906	93.2	0.884	87.7	0.801	90.3	0.834	**95.8**	**0.919**
SR	96.4	0.947	**98.8**	**0.983**	97.6	0.964	**98.8**	**0.983**	97.6	0.964
Avg.	90.8	0.831	91.6	0.826	88.6	0.786	90.1	0.802	**92.5**	**0.858**

Table 10 shows the comparison between running time taken by our method and other methods. There is no single method among these that can perform faster than our method. It clearly shows that kernelPLS is faster than the other algorithms. For example for AMLALL dataset time consumed by our method is 0.0891 s while time spent by ReliefF, mRMR, SVMrfe and PLS are 5.1510 s, 52.5854 s, 210.4046 s, 12.1222 s, respectively.

## Discussion

In this article, we proposed an effective multivariate-based feature filter method for cancer classification, namely, kernelPLS-based filter method. We showed that gene-gene interactions cannot be ignored in feature selection techniques to improve classification performance. In other words the nonlinear relationship of gene-gene interactions is a vital concept that can be taken into account to enhance accuracy. To capture these nonlinear relations of interaction between genes we used kernel method because kernel method can be used to reveal the intrinsic relationships that are hidden in the raw data. In order to capture the reasonable number of components, we make use of the relationship between PLS and linear discriminant analysis to determine the number of components in kernel space based on kernel linear discriminant analysis. To verify the importance of gene-gene interactions we compared our feature selector with other multivariate-based feature selection methods by using two classifiers SVM and KNN. Experimental results, expressed as both accuracy(Acc) and area under the ROC curve(AUC), showed that our method leads to promising improvement in ACC and AUC. We can conclude that the gene-gene interactions whats more, nonlinear relationships of gene-gene interactions are core interactions that can improve classification accuracy, efficiently. We can summarize the characteristics of proposed approach as follows: (1)Fast and efficient. The time complexity of deflation procedure used after the extraction of each component scale is 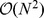, where 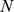 is the number of sample. In most cases, the number of sample in microarray data is less than 150, therefore, the running speed of kernelPLS procedure(feature selection time) is faster than others, which are summarized in table 10. (2)Model-free, e.g. no need the distributional assumptions. Because of small sample size, it is difficult to validate distributional assumptions, such as Gaussian distribution, Gamma distribution etc. (3)Applicable to both two-class as well as multi-class classification problems.

**Table 10:** The running time(s) of five feature filtering methods on two groups cancer classification datasets.

Class	Dataset	ReliefF	mRMR^1^	SVMrfe	PLS	kernelPLS
Two-class	A	5.1510	52.5854	210.4046	12.1222	**0.0891**
	B	5.1496	88.6176	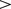 1e+003	10.6423	**0.1092**
	L	7.5420	52.8977	693.1857	16.8629	**0.2410**
	D	5.5614	53.1088	221.2261	12.0526	**0.0965**
	M	5.1343	51.9969	421.8250	19.2384	**0.2676**
	P	18.1848	65.1076	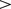 1e+003	64.2148	**0.6010**
Multi-class	St	34.0030	67.5321	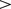 1e+003	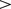 1e+003	**2.1180**
	Ly	2.7332	5.7846	217.2568	27.9456	**0.2361**
	Lu	10.2526	9.7816	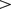 1e+003	17.8940	**0.5500**
	ML	6.6426	8.7484	791.0244	98.8890	**0.2586**
	SR	1.8230	5.8336	87.6536	8.8784	**0.1714**

1 Time required for selecting 1000 features.

In our method, the choice of kernel functions can affect the results. When high dimensionality exist(such as microarray datasets), the performance of linear kernel is better than Gauss kernel for our method. What's more, in case of linear kernel there is no noticeable effect on the results while adjusting its parameters.
